# Complex Structure and Biochemical Characterization of the *Staphylococcus aureus* Cyclic Diadenylate Monophosphate (c-di-AMP)-binding Protein PstA, the Founding Member of a New Signal Transduction Protein Family*

**DOI:** 10.1074/jbc.M114.621789

**Published:** 2014-12-11

**Authors:** Ivan Campeotto, Yong Zhang, Miroslav G. Mladenov, Paul S. Freemont, Angelika Gründling

**Affiliations:** From the ‡ Section of Microbiology and MRC Centre for Molecular Bacteriology and Infection,; § Section of Structural Biology, Department of Medicine, Imperial College London, London SW7 2AZ, United Kingdom

**Keywords:** Bacterial Signal Transduction, Bioinformatics, Crystal Structure, Nucleotide, Staphylococcus aureus (S. aureus), Complex

## Abstract

Signaling nucleotides are integral parts of signal transduction systems allowing bacteria to cope with and rapidly respond to changes in the environment. The *Staphylococcus aureus* PII-like signal transduction protein PstA was recently identified as a cyclic diadenylate monophosphate (c-di-AMP)-binding protein. Here, we present the crystal structures of the apo- and c-di-AMP-bound PstA protein, which is trimeric in solution as well as in the crystals. The structures combined with detailed bioinformatics analysis revealed that the protein belongs to a new family of proteins with a similar core fold but with distinct features to classical PII proteins, which usually function in nitrogen metabolism pathways in bacteria. The complex structure revealed three identical c-di-AMP-binding sites per trimer with each binding site at a monomer-monomer interface. Although distinctly different from other cyclic-di-nucleotide-binding sites, as the half-binding sites are not symmetrical, the complex structure also highlighted common features for c-di-AMP-binding sites. A comparison between the apo and complex structures revealed a series of conformational changes that result in the ordering of two anti-parallel β-strands that protrude from each monomer and allowed us to propose a mechanism on how the PstA protein functions as a signaling transduction protein.

## Teaser

**Background:** PstA is a cyclic di-AMP receptor and PII-like signal transduction protein.

**Results:** Apo-PstA and complex crystal structures reveal a novel cyclic di-AMP-binding mode and induced conformational changes.

**Conclusion:** PstA has a similar fold but distinct signal transduction properties from classic PII proteins, which function in nitrogen metabolism.

**Significance:** Identification of common features allows for rational prediction of cyclic di-AMP-binding sites.

## Introduction

Bacteria have evolved intricate signal transduction systems to detect and rapidly respond to changes in the environment. These systems include two-component systems, which normally consist of a membrane-embedded histidine kinase that senses an environmental stimulus and transduces this signal to an intracellular response regulator that coordinates an appropriate transcriptional response (1). In addition, various intracellular signal transduction proteins or domains within larger proteins can directly bind intracellular metabolites and adjust their activities or that of binding partner effector proteins very rapidly without the need of *de novo* protein synthesis. Other molecules, which have emerged as key components in signal transduction systems, are signaling nucleotides (2). In particular, cyclic dinucleotides have gained increased attention during recent years partly due to the discovery of novel nucleotides such as cyclic diadenylate monophosphate (c-di-AMP)^4^ and cyclic adenylate guanylate monophosphate (c-AMP-GMP) (3–6).

c-di-AMP is a widely distributed signaling nucleotide among the Gram-positive Firmicutes and Actinobacteria (7, 8). The stimuli leading to the production of c-di-AMP are currently not well understood. However, recent genome-wide protein-nucleotide interaction studies have led to the identification of several c-di-AMP-specific target proteins, providing the first clues toward pathways that are controlled by c-di-AMP. Currently known c-di-AMP-binding proteins include the cytoplasmic gating component KtrA/C of the Ktr potassium transport system, the histidine kinase KdpD, part of a two-component system that controls the expression of the Kdp potassium transport system, and CpaA, a predicted cation-proton antiporter, all identified in *Staphylococcus aureus* (9). Another c-di-AMP-binding protein is DarR, a transcription factor discovered in *Mycobacterium smegmatis* and suggested to be involved in the regulation of the expression of genes involved in fatty acid biosynthesis (10). Recently, c-di-AMP has also been described as an allosteric inhibitor of the *Listeria monocytogenes* pyruvate carboxylase, which is an essential metabolic enzyme that converts pyruvate to oxaloacetate (11). In the same work, four other hypothetical proteins were identified as c-di-AMP-binding proteins (11). One of these proteins of unknown function has also been previously identified as a c-di-AMP-binding protein in *S. aureus* and named PstA, based on a bioinformatics analysis and its annotation as a PII-like signal transduction protein (9).

The *S. aureus* PstA protein is the focus of this study. Using a structural, mutagenesis, and biochemical approach, we aimed to provide a better understanding on its function. Specifically, we aimed to determine whether this protein has all necessary features to serve as *bona fide* signal transduction proteins and how the interaction with c-di-AMP could lead to a signaling output. Very limited information is currently available on PII-like signal proteins such as PstA; however, much is known about prototypical PII proteins. These classical PII proteins are highly conserved and are some of the most widely distributed signal transduction proteins in nature, and they play a key role in nitrogen metabolism in bacteria and archaea (12–14). PII proteins function as trimers and through protein-protein interactions control the activity of a number of different proteins ranging from enzymes, transcription factors to membrane transporters. The ability of PII proteins to interact with their effector proteins is regulated through post-translational modifications as well as the binding of ligands, including ADP, ATP, Mg^2+^, and 2-oxoglutarate (2-OG), where 2-OG acts as a signal of the nitrogen availability in cell (15).

Based on sequence and operon structure, classical PII proteins have been grouped into the GlnB/K, the NifI, and an uncharacterized PII-New group (16). PII-like signal transduction proteins such as PstA share too little sequence similarity with these classical PII proteins and have not been included in previous bioinformatics analyses. The best characterized PII proteins belong to the GlnB/K group, which includes the closely related GlnB and GlnK proteins. Crystal structures of several GlnB and GlnK proteins with and without bound ligand have been reported (17–22). These studies revealed that PII proteins form homotrimers and have a highly conserved three-dimensional structure. Each monomer has a compact core made up of two α-helices and four β-strands, which are connected by so called T- and B-loops (13, 15). PII proteins interact through their T-loop with effector proteins, and in the absence of this protein-protein interaction, the T-loop is very flexible and is often unresolved in crystal structures (13, 15). The smaller B-loop contains a Walker A motif typical for an ATP-binding site (21). Under low nitrogen conditions, when the cellular 2-OG pool is high, PII proteins cooperatively bind 2-OG, ATP, and a Mg^2+^ ion. Two highly conserved residues, Gln-39 and Lys-58, which are located at the beginning and end of the T-loop, respectively, play a key role in ligand binding. When the 2-OG pool drops under high nitrogen conditions, PII proteins switch to the ADP-bound state with residues Gln-39 and Lys-58 now forming a salt bridge instead of interacting with the ligands (23–25). This results in the T-loop adopting two different conformations depending on the cellular 2-OG concentration, allowing PII proteins to interact or disengage with their specific effector proteins.

The binding of 2-OG is therefore a key aspect of the sensory capacity of classical PII signal transduction proteins. However, an exception has been reported for a member of the GlnB/K group, which does not appear to bind 2-OG, and hence might sense and respond to different signals (25). Also, the effector binding properties of the PII-New group are currently not known. Finally, the recent identification of the PII-like signal transduction proteins in *S. aureus* and *L. monocytogenes* as c-di-AMP-binding proteins indicates that the processes controlled by PII-like proteins and the regulatory molecules these proteins respond to are even more complex than previously anticipated.

To elucidate the signal transduction capacity of the *S. aureus* PstA protein as well as provide information on the mode of c-di-AMP binding and its functional consequence, we performed a structural, biochemical, and mutagenesis analysis. Our analyses revealed that PstA proteins are clearly distinct from classical and previously characterized PII proteins and provide a rationale for the c-di-AMP binding in PstA *versus* the ATP, Mg^2+^, and 2-OG binding in PII proteins. The co-complex crystal structure between c-di-AMP and the *S. aureus* PstA protein not only revealed the nucleotide-protein interactions but a comparison with the apo-PstA structure allowed us to suggest a mechanism for the signal transduction properties of this protein.

## EXPERIMENTAL PROCEDURES

### 

#### 

##### Strain and Plasmid Constructions

Bacterial strains and primers used in this study are listed in Tables 1 and 2, respectively. *Escherichia coli* strains were grown in Luria Bertani (LB) medium. When appropriate, cultures were supplemented with antibiotics as indicated in Table 1. The construction of plasmid pET28b-His-*pstA*, for the expression and purification of N-terminally His-tagged PstA (His-PstA_SA_) protein from *S. aureus,* has been described in a previous study (9). pET28b-His-*pstA* was used as a template in PCRs for the construction of the plasmids used for the expression of the different His-PstA_SA_ variants with alanine substitutions or amino acid deletions. Specifically, for construction of plasmid pET28b-His-*pstA*(T28A), primer pairs ANG1591/ANG1875 and ANG1876/ANG1592 were used in the first round of PCR. The resulting products were gel-purified and fused using primer pair ANG1591/ANG1592 in a second round of PCR. The final product was digested with enzymes NdeI and EcoRI and ligated with plasmid pET28b that has been cut with the same enzymes. Plasmids pET28b-His-*pstA*(GGFL-AAAA), pET28b-His-*pstA*(N41A), pET28b-His-*pstA*(T43A), pET28b-His-*pstA*(R67A), and pET28b-His-*pstA*(Δd71–87), were constructed in a similar manner replacing primers ANG1875/ANG1876 with primers ANG1877/ANG1878, ANG1879/ANG1880, ANG1881/ANG1882, ANG1883/ANG1884, and ANG1886/ANG1887, respectively. For construction of plasmid pET28b-His-*pstA*(K2A), primer pair ANG1931/ANG1592 was used. The resulting products were digested and ligated with cut pET28b plasmid as described above. All plasmids were initially recovered in *E. coli* strain XL-1 Blue, and sequences of inserts were confirmed by fluorescent automated sequencing (GATC Biotech). For protein expression and purification, the plasmids were introduced in *E. coli* strain BL21(DE3) (Table 1).

**TABLE 1:** **Bacterial strains used in this study**

Strain	Relevant features	Ref.
***E. coli* strains**		
XL1 Blue	Cloning strain, TetR--ANG127	Stratagene
BL21(DE3)	*E. coli* strain used for protein expression--ANG191	Novagen
ANG1824	pET28b in XL1 Blue; KanR	Novagen
ANG1867	pET28b in BL21 (DE3); KanR	Novagen
ANG2702	pET28b-His-*pstA* in XL1 Blue; KanR	9
ANG2711	pET28b-His-*pstA* in BL21 (DE3); KanR	9
ANG3043	pET28b-disA_BT_-His in XL1 Blue; KanR	This study
ANG3048	pET28b-disA_BT_-His in BL21 (DE3); KanR	This study
ANG3352	pET28b-His-*pstA (*K2A) in XL1 Blue; KanR	This study
ANG3278	pET28b-His-*pstA* (D12A) in XL1 Blue; KanR	This study
ANG3280	pET28b-His-*pstA* (T28A) in XL1 Blue; KanR	This study
ANG3279	pET28b-His-*pstA* (GGFL-AAAA) in XL1 Blue; KanR	This study
ANG3282	pET28b-His-*pstA* (N41A) in XL1 Blue; KanR	This study
ANG3283	pET28b-His-*pstA* (T43A) in XL1 Blue; KanR	This study
ANG3285	pET28b-His-*pstA* (R67A) in XL1 Blue; KanR	This study
ANG3286	pET28b-His-*pstA* (Δ71–87) in XL1 Blue; KanR	This study
ANG3353	pET28b-His-*pstA* (K2A) in BL21 (DE3); KanR	This study
ANG3292	pET28b-His-*pstA* (D12A) in BL21 (DE3); KanR	This study
ANG3293	pET28b-His-*pstA* (T28A) in BL21 (DE3); KanR	This study
ANG3296	pET28b-His-*pstA* (GGFL-AAAA) in BL21 (DE3); KanR	This study
ANG3294	pET28b-His-*pstA* (N41A) in BL21 (DE3); KanR	This study
ANG3295	pET28b-His-*pstA* (T43A) in BL21 (DE3); KanR	This study
ANG3297	pET28b-His-*pstA* (R67A) in BL21 (DE3); KanR	This study
ANG3298	pET28b-His-*pstA* (Δ71–87) in BL21 (DE3); KanR	This study
**Other strains**		
BMB171	*B. thuringiensis* BMB171– NG3001	27

**TABLE 2:** **Primers used in this study** Restriction sites are underlined.

Number	Name	Sequence
ANG1591	F-NdeI-PstA	GGGCATATGAAAATGATTATAGCGATCGTACAAG
ANG1592	R-EcoRI-PstA	GGGGAATTCTTAAAATTGATGGAATGCATCAAC
ANG1711	5-NcoI-DisA-Bt	CATGCCATGGAAGAAAATAAGCAACGTGTCAAAAG
ANG1712	3-XhoI-DisA-Bt	CCGCTCGAGATTGTGTCTACTCATATAGAGATGCTCTTG
ANG1872	H6.pstA (D12A)-F	GGAATTCCATATGAAAATGATTATAGCGATCGTACAAGATCAAGCTAGTCAGGAACTTGC
ANG1875	H6.pstA (T28A)-R	GTTGTTGCCAATTTTGCTGCTCTAAAGTTATTTTTAAC
ANG1876	H6.pstA (T28A)-F	CAGCAAAATTGGCAACAACAGGTGG
ANG1877	H6.pstA (GGFL-AAAA)-R	TTGCAGCCGCAGCTGTTGTTGCCAATTTTGTTG
ANG1878	H6.pstA (GGFL-AAAA)-F	CAGCTGCGGCTGCAAGAGCGGGTAATACAACATTC
ANG1879	H6.pstA (N41A)-R	GCACCCGCTCTTAAAAACCCAC
ANG1880	H6.pstA (N41A)-F	TTTAAGAGCGGGTGCTACAACATTCTTATGTGGTGTC
ANG1881	H6.pstA (T43A)-R	TGCTGTATTACCCGCTCTTAAAAACC
ANG1882	H6.pstA (T43A)-F	GCGGGTAATACAGCATTCTTATGTGGTGTCAATG
ANG1883	H6.pstA (R67A)-R	TCTGCATTACCACACGTTTGATTAATC
ANG1884	H6.pstA (R67A)-F	GTGTGGTAATGCAGAACAGTTGGTTTCACC
ANG1886	H6.pstA (Δ71–87)-R	CAACTGTTCTCTATTACCACACGTTTG
ANG1887	H6.pstA (Δ71–87)-F	AATAGAGAACAGTTGCCAGTTGAAGTTGAAGTTGG
ANG1931	H6.pstA (K2A)-F	GGAATTCCATATGGCAATGATTATAGCGATCGTACAAGATCAAG

Plasmid pET28b-*disA*_BT_-His was produced for the expression and purification of the C-terminally His-tagged c-di-AMP cyclase DisA from *Bacillus thuringiensis* (DisA_BT_-His) using a strategy similar to that described by Zheng *et al.* (26). To this end, the *disA* gene was amplified from *B. thuringiensis* BMB171 (27) chromosomal DNA by PCR using primer pair ANG1711/1712. The resulting product was cut with restriction enzymes NcoI and XhoI and ligated with plasmid pET28b, which has been cut with the same enzymes. The resulting plasmid, pET28b-*disA*_BT_-His, was initially obtained in *E. coli* strain XL-1 Blue yielding strain ANG3043. For protein expression, the plasmid was introduced into strain BL21(DE3) yielding strain ANG3048.

##### Protein Expression and Purification

*E. coli* BL21(DE3) strains (Table 1) were used for the expression and purification of the N-terminally His-tagged PstA (His-PstA_SA_) protein and the different His-PstA variants. 1-liter cultures of the different strains were grown at 37 °C to an *A*_600_ of 0.5 to 0.7, and protein expression was induced with 0.5 mm isopropyl 1-thio-β-d-galactopyranoside (IPTG) and cultures were incubated overnight at 16 or 18 °C. Protein purifications by Ni-NTA affinity chromatography were performed as previously described with the exception that 10 mm imidazole was included in the lysis buffer (28). Proteins were further purified by size exclusion chromatography using a 50 mm Tris-HCl, pH 7.5, 200 mm NaCl, 5% glycerol buffer system. Protein containing fractions were pooled and concentrated to around 3 mg/ml using 10-kDa cutoff filtration devices. For crystallization studies, a 20 mm Tris-HCl, pH 7.5, buffer was used for the size exclusion chromatography and the protein was concentrated to ∼10 mg/ml. Protein concentrations were determined using the BCA Protein assay kit from Pierce. The purity of the purified proteins was assessed on Coomassie stained gels following separation of the indicated amount of protein on 15% (w/v) SDS-polyacrylamide gels.

##### Protein Crystallization and Structure Determination

For crystallization, sitting-drop trials were set up by robot (Mosquito, TTP Labtech) in 96-wellplates using 1440 different commercial conditions (Molecular Dimension: MemStart, MemSys, PACT premier, JCSG-plus, MemGold, ProPlex, PGA Screen; Hampton Research: Crystal Screen, PEG/Ion Screen 1 and 2, Natrix, Index, SaltRx; Rigaku: Wizard Classic 1, 2 and 3; Jena Bioscience: JBScreen Cryo HTS) with 50 μl solution per well and a 200 nl drop size (100 nl reservoir and 100 nl protein solution, at 20 °C). The crystals of His-PstA_SA_ appeared in 2 to 3 days at 20 °C under four different conditions: 100 mm sodium citrate, pH 4.6, 0.2 m KCl, 37% pentaerythritol propoxylate (condition 1), 100 mm sodium acetate pH 4.6, 30% MPD (condition 2), 200 mm Na malonate pH 4.6 and 20% PEG3350 (condition 3), 200 mm succinic acid, pH 6.8 (condition 4). The best crystals were obtained in condition 3. Data collection was performed at 100K at the SOLEIL synchrotron, beamline PROXIMA 1 from a single crystal grown in condition 3. The His-PstA_SA_ crystals belonged to space group P321 with unit cell parameters a = 56.75Å, b = 56.75Å, c = 66.17Å, α = β = 90°, γ = 120°.

Data were indexed in XDS (29), scaled in SCALA (30) and R_free_ was generated randomly in UNIQUE (31). Molecular replacement was performed in PHENIX (32) using an ensemble of each monomer of the structure of *Pediococcus pentosaceus* ATCC 25745 protein PEPE_1480 (PDB code 3M05), a PstA homolog, as a search model. Two datasets were collected from the same crystal and merged together to obtain high multiplicity for sulfur phasing (total rotation ϕ = 360°). However anomalous signal extended only to 4.5Å hampering direct experimental phasing. Nevertheless, anomalous maps were generated in SFALLS (31) and superposed on the MR solution to guide model building starting from the sulfur-containing amino acids, followed by the building of aromatic amino acids in COOT (33). Ten cycles of rigid body refinement (10.0–6.0Å) were followed by 10 cycles of restrained body refinement (49.18–2.8Å) in REFMAC (34) leading to R_factor_ = 0.25 R_free_ = 0.29. TLS group analysis was performed using the TLSMD-server (35) and performed in REFMAC. Model building was reiterated in COOT and structure validation was performed in MOLPROBITY (36). Final statistics for data collection and model refinement are presented in Table 3. The structure-based sequence alignment was generated in PyMOL and analyzed with JALVIEW (37). Structural homology searches were performed using the DALI server (38) and amino acid conservation was mapped onto the crystal structure using ConSurf (39).

**TABLE 3:** **Crystallographic data and refinement statistics**

	PstA apo	PstA + c-di-AMP
Beamline	PROXIMA1	PROXIMA2
Space group	*P321*	*I121*
**Cell parameters**		
*a*, *b*, *c* (Å),	56.75, 56.75, 66.17,	64.21, 65.65, 85.16
α, β, γ (^o^)	90.00, 90.00, 120.00	90.00, 95.98, 90.00
Wavelength (Å)	1.82	0.98
Resolution (Å)	49.12-3.00 (3.16-3.00)	42.35-2.00 (2.05-2.00)
*R*_merge_	0.109 (0.556)	0.083 (0.446)
*R*_pim_ (all *I*^+^ and *I*^−^)	0.018 (0.090)	0.057 (0.293)
〈*I*〉/S.D. 〈*I*〉	27.2 (7.9)	9.6 (2.9)
Completeness (%)	100.0 (100.0)	99.2 (99.8)
Redundancy	38.2 (38.7)	3.5 (3.5)
Wilson B factor (Å^2^)	82.7	22.1
No. of reflections	102,187 (14500)	82,553 (6093)
No. of unique	2672 (375)	23,649 (1739)
CC (1/2)	0.999 (0.987)	0.996 (0.862)
*R*_factor_	0.246 (0.385)	0.188 (0.243)
*R*_free_	0.293 (0.485)	0.232 (0.261)
No. of atoms	605	2780
Protein	605	2474
Waters		174
c-di-AMP		132
Average B-factors (Å^2^)		
Protein	47.1	23.6
Water		32.2
c-di-AMP		16.6
Root mean square deviations		
Bond lengths (Å)	0.011	0.014
Bond angles (°)	1.28	1.82
Ramachandran most favored ( %)	97	100
Ramachandran allowed	3	0
PDB code	4D3G	4D3H

For co-crystallization studies, His-PstA_SA_ at a concentration of 10 mg/ml was incubated in 20 mm Tris-HCl, pH 7.5, 10 mm MgCl_2_ buffer containing 4 mm c-di-AMP (InvivoGen) for 30 min on ice and subsequent crystallization trials were performed in condition 3, analogous to the apo-protein. Data collection was performed from a single crystal at 100K at SOLEIL synchrotron, beam line PROXIMA2. The crystals belonged to space group *I*121 with unit cell parameters: a = 64.21Å, b = 65.65Å, c = 85.16Å, α = 90°, β = 95.98°, γ = 90°. The structure was solved by molecular replacement using the apo-structure as a model in PHASER (40). Model building, refinement and validation were performed as described for the apo-structure. The final R_factor_ and R_free_ were 0.19 and 0.23, respectively. Superposition of the apo and complex structures was performed with SUPERPOSE (31). The structures were deposit in the protein data bank under PDB codes 4D3G (apo PstA) and 4D3H (c-di-AMP/PstA complex).

##### Preparation of E. coli Whole Cell Lysates

For the preparation of whole cell lysates, *E. coli* BL21(DE3) strains containing the empty pET28b vector, pET28b-His-*pstA* or derivatives thereof for the expression of the different His-PstA_SA_ variants, were grown overnight at 30 °C in LB medium. The next morning, the protein expressed was induced with 1 mm isopropyl 1-thio-β-d-galactopyranoside, and the cultures were incubated for a further 6 h at 30 °C. Bacteria from 1 ml of culture were collected by centrifugation and suspended to an *A*_600_ of 40 in binding buffer (40 mm Tris, pH 7.5, 100 mm NaCl, 10 mm MgCl_2_), containing 2 mm PMSF, 20 μg/ml DNase, and 0.5 mg/ml lysozyme. Cells were lysed by three freeze and thaw cycles and subsequently used in binding assays or stored at −20 °C.

##### Synthesis of ^32^P-Labeled c-di-AMP

^32^P-Labeled c-di-AMP was synthesized from [α-^32^P]ATP (PerkinElmer Life Sciences) by incubating 55.5 nm [α-^32^P]ATP with 2 μm purified *B. thurengensis* DisA_BT_-His protein in 40 mm Tris, pH 7.5, 100 mm NaCl, 10 mm MgCl_2_ binding buffer at 45 °C overnight. The sample was subsequently incubated for 10 min at 95 °C, and the DisA_BT_-His protein was removed by centrifugation at 17,000 × *g* for 5 min; the supernatant was transferred to a new tube and stored at −20 °C. The conversion of [α-^32^P]ATP to ^32^P-labeled c-di-AMP was estimated to be 87–96%, as assessed by TLC and densitometry analysis, which was performed as described previously (9).

##### Differential Radial Capillary Action of Ligand Assay (DRaCALA)

c-di-AMP binding to His-PstA_SA_ or its variants was performed with DRaCALAs, the principle of which is described in Roelofs *et al.* (41) and was adapted by Corrigan *et al.* (9) to study c-di-AMP protein interactions. Briefly, *E. coli* whole cell lysates or 10 μm solutions of purified protein (for standard assays) in binding buffer were mixed with ∼1 nm ^32^P-labeled c-di-AMP and incubated at room temperature for 5 min. Then, 2.5 μl of these reactions were spotted onto nitrocellulose membranes (Hybond-ECL, GE Healthcare) and air-dried, and radioactivity signals were detected and quantified as described previously (9, 41). For *K_d_* measurement, 2-fold serial dilutions of purified His-PstA_SA_ or the different variants were prepared in binding buffer (40 mm Tris, pH 7.5, 100 mm NaCl, 10 mm MgCl_2_) at a starting concentration of 300 μm and subsequently mixed with ∼1 nm ^32^P-labeled c-di-AMP. The fractions of ligand bound and *K_d_* values were calculated as described previously (41). To determine *k*_off_ values, a 10 μm solution of purified His-PstA_SA_ protein in binding buffer was mixed with 1 nm ^32^P-labeled c-di-AMP and incubated at room temperature for 5 min. Then, 2.5 μl of these reactions were spotted onto nitrocellulose membranes and set as time 0. Afterward, cold c-di-AMP at a final concentration of 300 μm was added to the remaining sample, and after incubation for 10, 20, 30, 40, 50, 60, 90, 120, and 180 s, 2.5 μl of these reaction were spotted onto a nitrocellulose membrane. The fraction of ligand bound and *k*_off_ values were calculated as described previously (41).

##### Multiangle Laser Light Scattering (SEC-MALs) Analysis

Purified His-PstA_SA_ protein was adjusted to a concentration of 100 μm in 50 mm Tris-HCl, pH 7.5, 200 mm NaCl, and 5% glycerol buffer. One hundred microliters of this solution was subjected to a size exclusion chromatography step using a Superdex 10/300 column, which was coupled to a multiangle laser light scattering detector (Wyatt Technology Corp.). The data were processed with the ASTRA 5.3.4 software and fitted according to the Zimm model for static light scattering. Experiments were repeated twice with two different protein preparations, and a representative graph is shown.

##### Bioinformatics Analysis

The PstA_SA_ protein sequence (SAUSA300_0460) was used as query sequence in a PSI-BLAST search using default settings to identify homologs in the NCBI nonredundant protein sequence database. Four iterations of the search were performed at which point only the number of classical PII proteins increased. This yielded 911 sequences with a maximum expect (*e*) values below 3e-04 and a minimum sequence coverage and sequence identity of 60 and 30%, respectively. These sequences were exported and used in JALVIEW (37) and a multisequence alignment generated after 10 iterations and a conserved logo-sequence with Cluster Omega (42).

## RESULTS

### 

#### 

##### Bioinformatics Analysis of PstA Proteins

**FIGURE 1.**
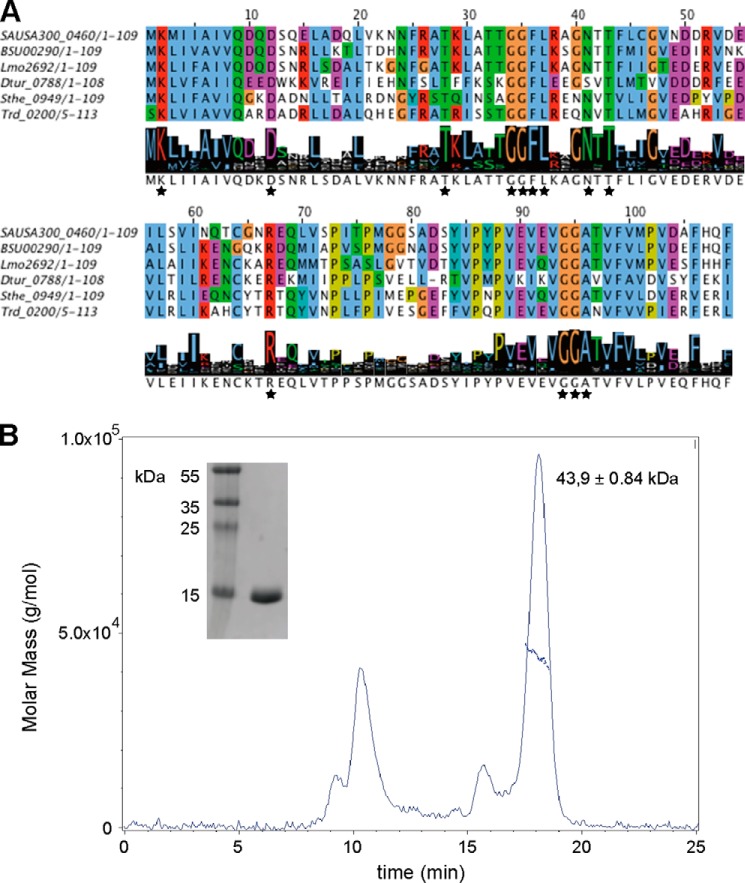
**Multiple sequence alignment of PstA_SA_ homologs and PstA_SA_ oligomerization state.** *A,* 911 PstA_SA_ homologs were identified using a PSI-BLAST search and aligned with Cluster Omega. Exemplars for a few closely related family members (SAUSA300_0460, *S. aureus* subsp. *aureus* USA300_FPR3757 (CA-MRSA); BSU00290, *B. subtilis* subsp. *subtilis* 168; Lmo2692, *L. monocytogenes* EGD-e) and a few distantly related members (Dtur_0788, *Dictyoglomus turgidum*; Sthe_0949, *Sphaerobacter thermophiles*; Trd_0200, *Thermomicrobium roseum*) are shown along with the logo motif produced for all PstA_SA_ homologs. The full alignment can be obtained from the authors. Highly conserved amino acids are indicated with *black asterisks. B,* SEC-MALs analysis of purified His-PstA_SA_. A 100 μm solution of purified His_6_-PstA_SA_ protein was analyzed by SEC-MALs and the molar mass and differential refractive index plotted *versus* the elution volume from a Superdex-200 10/300 column. The purified His_6_-PstA_SA_ protein (calculated mass of 13.9 kDa) eluted as a 43.9-kDa complex indicating that the protein forms a trimer in solution. Of note, the first peak is likely aggregated protein, which eluted in the void volume of the column. *Inset* on the *left* shows a Coomassie-stained gel with 1 μg of purified protein and molecular mass marker proteins with sizes shown in kDa.

PstA, a PII-like signal transduction protein of unknown function, was identified as a c-di-AMP receptor protein in both *S. aureus* and *L. monocytogenes* (9, 11). To gain insight into the phylogenetic distribution of PstA-type proteins and define common and unique features compared with classical PII proteins, a detailed bioinformatics analysis was carried out. A PSI-BLAST search was performed using the PstA protein SAUSA300_0460 from *S. aureus* USA300_FPR3757 (from here on referred to as PstA_SA_) as query sequence. This yielded 911 sequences with maximum expect (*e*) values below 3e-04 and a minimum sequence coverage and sequence identity of 60 and 30%, respectively. The majority of the retrieved sequences was annotated as hypothetical proteins, although several were annotated as nitrogen regulatory PII-related proteins. Next, a multiple sequence alignment was performed with the 911 PstA-like sequences with Cluster Omega and 10 iterations, until the alignment was stable and a consensus logo motif was generated (Fig. 1*A*). This analysis revealed that amino acids Lys-2, Asp-12, and Arg-67 (using PstA_SA_ protein amino acid numbers) were highly conserved and present in more than 95% of the PstA sequences (Fig. 1*A*). In addition, highly conserved amino acid motifs were identified between residues Thr-28 and Thr-43 with the consensus sequence of TKL*XXX*GGFL*XX*GNTT and a highly conserved ^94^GGA^96^ amino acid stretch toward the C-terminal end of the protein (Fig. 1*A*). A segment of ∼30 amino acids between the conserved residues Arg-67 and Gly-94 showed the highest sequence variation; as discussed later, this region is likely involved in effector protein binding (Fig. 1*A*).

##### Apo Structure of PstA_SA_

**FIGURE 2.**
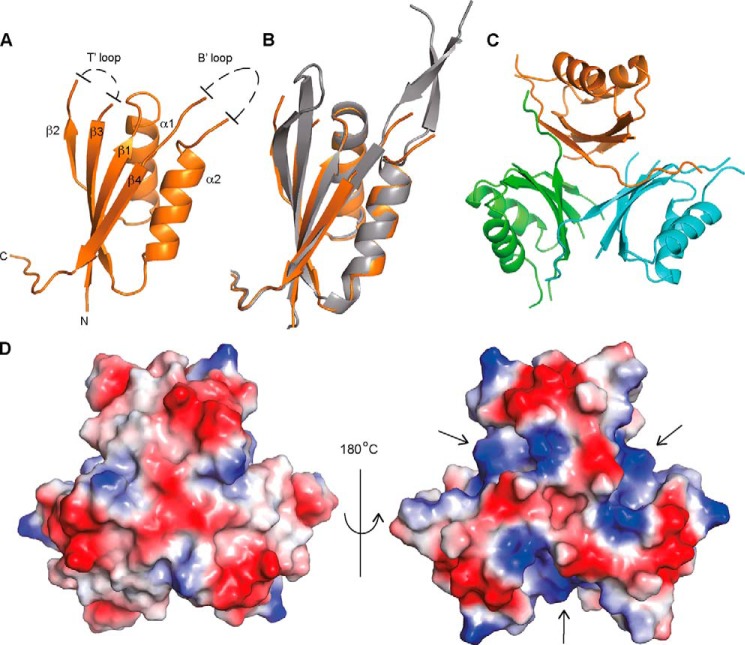
**Crystal structure of apo-PstA_SA_.** *A,* schematic representation of the crystal structures of a PstA_SA_ monomer with α-helices and β-strands numbered, and unstructured T′- and B′-loops schematically indicated; *B*, overlay between PstA_SA_ (*orange*) and the *P. pentosacues* ATCC 25745 protein PEPE_1480 (PDB code 3M05) (*gray*); and *C*, PstA_SA_ trimer with the individual monomers shown in different colors. *D,* electrostatic surface potential representation (*blue,* positive potential; *red,* negative potential) of the PstA_SA_ trimer shown on the *left* in the same orientation as in schematic representation in *C* and on the *right* rotated by +180° around the *y* axis.

To further characterize the PstA_SA_ protein, we pursued the determination of its structure. The N-terminally His-tagged PstA_SA_ protein (His-PstA_SA_) was expressed and purified from *E. coli* strain BL21(DE3) (Fig. 1*B*). The His-PstA_SA_ protein has a calculated mass of 13.9 kDa; however, SEC-MALs analysis revealed that the protein forms a 43.9 ± 0.84-kDa complex in solution (Fig. 1*B*) suggesting that PstA_SA_ is trimeric in solution, similar to classical PII proteins. The crystal structure of the *S. aureus* PstA proteins was determined at 3.0 Å resolution by molecular replacement using the structure of the *Pediococcus pentosaceus* ATCC 25745 protein PEPE_1480 (PDB code 3M05) as a model (Fig. 2, *A* and *B*). This protein, which is also of unknown function, shares 48% sequence identity with PstA_SA_. Although the PstA_SA_ protein crystallized as a monomer in the asymmetric unit, upon inspection of the crystal packing it became clear that the protein forms trimers within the crystal lattice consistent with a trimeric state in solution (Fig. 2, *C* and *D*). The PstA_SA_ monomer includes a core of four β-strands and two α-helices connected by two highly flexible loops (Fig. 2, *A* and *B*). The amino acids from 34 to 40, which following the nomenclature for PII proteins will be referred to as T′-loop residues, and residues 68 to 92, which will be referred to as B′-loop residues, were not visible in the electron density maps indicating that these residues are disordered (Fig. 2*A*). The main inter-monomer contacts in the PstA_SA_ trimer are provided by the interaction of β_4_, from one monomer with the C terminus of the neighboring monomer, through a network of hydrogen bonds (Fig. 2*C*). Inspection of the surface potential of the trimer highlighted a large, accessible, and positively charged area at the monomer-monomer interface (Fig. 2*D*), which, similar to the recently reported structure of the *L. monocytogenes* pyruvate carboxylase enzyme in complex with c-di-AMP (11), could serve as a potential nucleotide-binding site.

##### Structural Comparison of PstA and Classical PII Proteins

**FIGURE 3.**
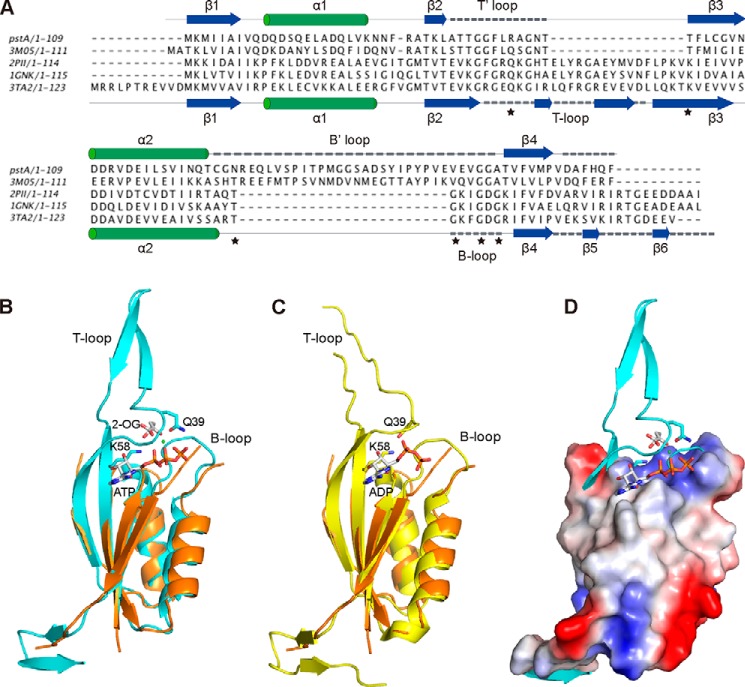
**Structural comparison of PstA and classical PII proteins.** *A,* structure-based alignment of PstA_SA_, *P. pentosacues* ATCC 25745 protein PEPE_1480 (PDB code 3M05), *E. coli* PII proteins GlnK (PDB code 1GNK), *E. coli* GlnB (PDB code 2PII), and *Archaeoglobus fulgidus* PII protein AF_1750 (PDB code 3TA2). The secondary structures for PstA_SA_ and 3TA2 are shown *above* and *below* the alignment, respectively, with T/T′- and B/B′-loops indicated with *dashed lines*. Also, the highly conserved PII protein residues Gln-39 and Lys-58 at the base of the T-loop as well as the highly conserved TG*XX*GDGKI motif within the B-loop of PII proteins are indicated with *asterisks. B,* overlay of the PstA_SA_ monomer structure (*orange*) in schematic representation with the monomer structure of the *A. fulgidus* PII protein AF_1750 in the ATP, Mg^2+^ (*green dot*), and bound to the 2-OG ligand (PDB code 3TA2); *C,* overlay with the monomer structure of the *A. fulgidus* PII protein AF_1750 in the ADP ligand-bound form (PDB code 3TA1) (24). T- and B-loops as well as highly conserved amino acids Gln-39 and Lys-58 (shown in *stick* representation) as well as ATP, 2-OG, and ADP ligands are labeled in *B* and *C. D,* overlay of the surface potential representation of the PstA_SA_ protein with the *A. fulgidus* PII protein AF_1750 and bound ligand in schematic representation.

Structural similarity searches using the DALI server identified PII proteins as close structural homologs to the PstA_SA_ protein. Structure-based alignments (Fig. 3*A*) and the superposition of PstA_SA_ with members of the PII proteins family revealed a high degree of structural similarity within the core fold of the protein but with considerable variation in the size and sequence of the loops (Fig. 3). Specifically, the large T-loop in PII proteins, which is involved in protein-protein interactions is replaced with a shorter T′-loop in PstA (Fig. 3*A*). Furthermore, the highly conserved residues Gln-39 and Lys-58 found in PII proteins at the beginning and the end of the T-loop, respectively, are absent in PstA-like proteins (Fig. 3). These two residues play a key role in the signal transduction function of PII proteins (15, 23, 24). They are involved in the binding of 2-OG/Mg^2+^/ATP/, at a high intracellular 2-OG concentration, although at a low 2-OG concentration when the PII protein is in the ADP-bound state, these two residues form a salt bridge, leading to a drastic change in the conformation of the T-loop (Fig. 3, *B* and *C*) (15, 23, 24). Furthermore, the B-loop of PII proteins is replaced in PstA by a larger loop that lacks the highly conserved B-loop motif TG*XX*GDGKI, which is essential for ATP binding in PII proteins (Fig. 3*A*). In summary, the comparative sequence and structural analyses suggest that PstA_SA_ belongs to a new family of proteins with a similar core fold but with distinct features to PII proteins. The biological function of PstA-like proteins is currently not known. However, the absence of key residues required for ATP, Mg^2+^, and 2-OG binding and hence the function of classical PII proteins suggest that PstA proteins have a cellular function distinct from classical PII proteins.

##### Interaction of PstA_SA_ with c-di-AMP

**FIGURE 4.**
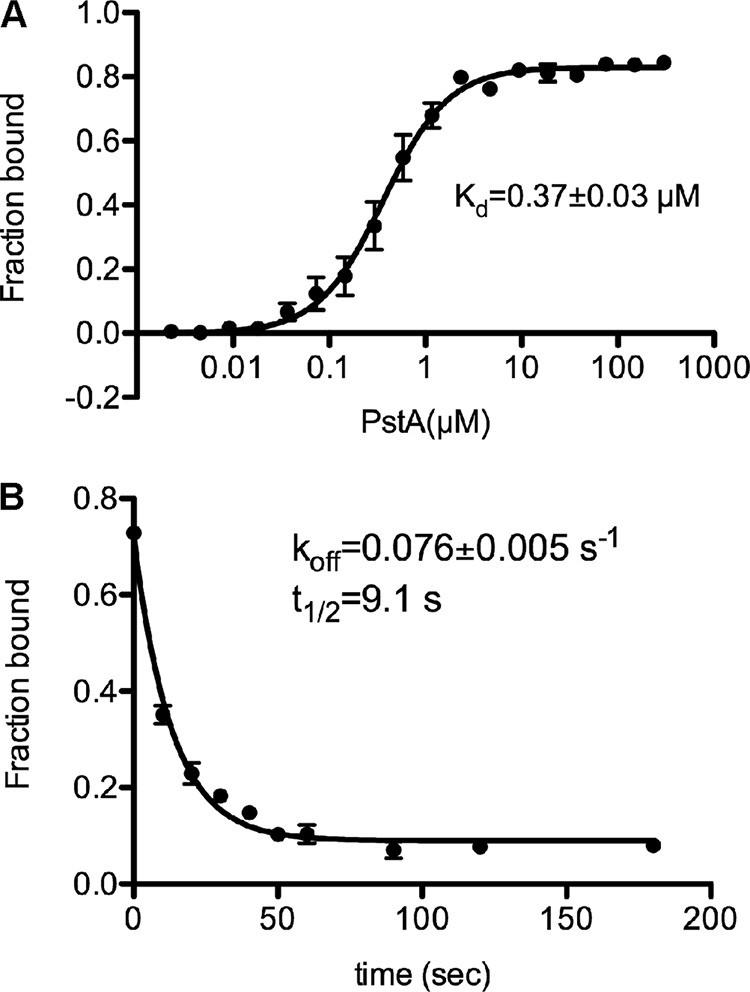
**Binding characteristics between c-di-AMP and PstA_SA_.** *A,* binding curve and *K_d_* determination for c-di-AMP and purified His-PstA_SA_. DRaCALAs were performed with ^32^P-labeled c-di-AMP and purified His-PstA_SA_ at a starting protein concentration of 300 μm, and fraction-bound and *K_d_* values were determined from the curve as described previously (41). The average values from three independent experiments are plotted with standard deviations. *B,* dissociation curve and *k*_off_ determination for c-di-AMP and purified His-PstA_SA_. A 10 μm solution of purified His-PstA was incubated with ^32^P-labeled c-di-AMP. At time 0, 300 μm cold c-di-AMP was added, and the displacement of ^32^P-labeled c-di-AMP was measured at 10-, 20-, 30-, 40-, 50-, 60-, 90-, 120-, and 180-s time points. Fraction-bound values were plotted, and the *k*_off_ value was determined as described previously (41). The average values from three independent experiments are plotted with standard deviations.

Previous work on PstA_SA_ has shown that the protein can bind c-di-AMP (9). This binding is specific as other nucleotides, including ATP, cyclic adenylate monophosphate (cAMP), or cyclic diguanylate monophosphate (c-di-GMP), could not compete for the binding, even when present in large excess over c-di-AMP (9). The intracellular c-di-AMP concentration in *S. aureus* is in the low micromolar range (9). To assess whether the interaction between c-di-AMP and PstA_SA_ is physiological, the *K_d_* value for this interaction was determined by the differential radial capillary action of ligand assay (DRaCALA) (41) using purified His-PstA_SA_ protein and ^32^P-labeled c-di-AMP. Using this method, an interaction with a *K_d_* of 0.37 ± 0.03 μm was measured (Fig. 4*A*). This interaction is in a physiologically relevant range and comparable with previously reported *K_d_* values for other c-di-AMP target proteins of *S. aureus* and *L. monocytogenes*, which were in the range of 0.1 to 8 μm (9–11). The *k*_off_ value for c-di-AMP and His-PstA_SA_ in the presence of 300 μm cold c-di-AMP was determined as 0.076 ± 0.005 s^−1^ (Fig. 4*B*), which corresponds to a half-life of 9.1 s, indicating a rapid equilibrium of bound and free protein.

##### PstA_SA_ Structure in Complex with c-di-AMP

**FIGURE 5.**
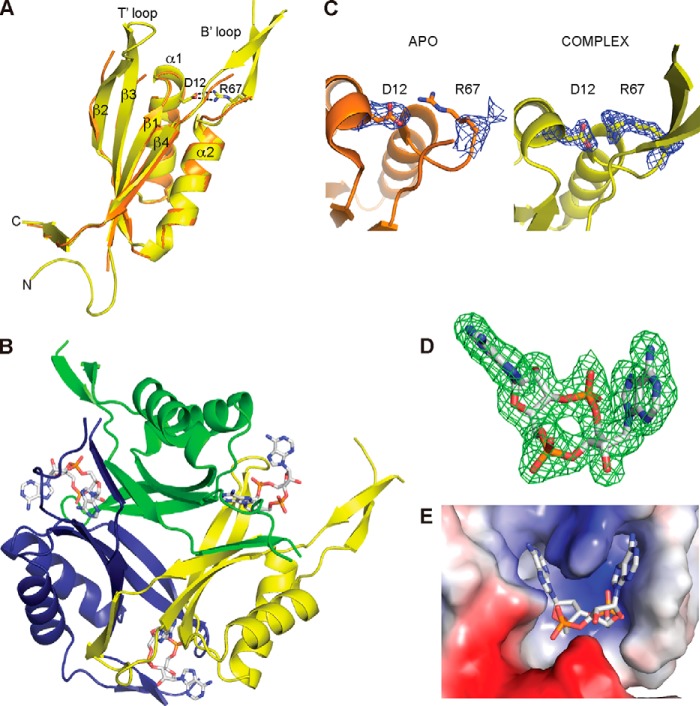
**PstA_SA-_c-di-AMP complex structure.** *A,* overlay of the apo-PstA (*orange*) and PstA_SA_·c-di-AMP complex (*yellow*) monomer structures in schematic representation. Amino acids Asp-12 and Arg-67 form a salt bridge in the complex structure and are shown as *sticks*; α-helices and β-sheets are numbered, and T′ and B′-loops are labeled. *B,* schematic representation of the c-di-AMP/PstA_SA_ trimer with individual monomers shown in different colors. *C,* close-up view of apo-PstA (*orange*) and complex (*yellow*) secondary structure elements with Asp-12 and Arg-67 residues shown as *sticks*, and the corresponding 2*F_o_* − *F_c_* map contoured in *blue* at 1.0 root mean square deviation. For the Arg-67 residue in the apo structure, only density for the C_β_ is present. *D,* simulated annealing composite omit map at 1.0 root mean square deviation of the bound c-di-AMP in monomer A. *E,* close-up view of a c-di-AMP-binding site at the monomer-monomer interface shown in surface potential representation.

To gain molecular insights into the nucleotide protein interactions, the c-di-AMP·PstA_SA_ complex structure was pursued. To this end, the His-PstA_SA_ protein was incubated with 4 mm c-di-AMP and subsequently co-crystallized. The protein crystallized as a trimer in the asymmetric unit, and the crystal structure of the protein·ligand complex was solved at 2.0Å resolution by molecular replacement using the apo structure as a model (Fig. 5*A*). The c-di-AMP bound at the monomer-monomer interface with three molecules bound per trimer (Fig. 5*B*). Superposition of the ligand·complex structure with the apo structure revealed no major changes within the core of the protein and a root mean square deviation (r.m.s.d.) for the Cα atoms of 0.9 Å for the trimer. The main differences between the unbound and bound structures was in the T′-loop, which was disordered in the apo structure but became structured in the c-di-AMP·PstA_SA_ complex, thereby providing many stabilizing interactions with the ligand, particularly through Phe-36 (see detailed description below). In addition, the large B′-loop (amino acids 68–92), which was unresolved in the apo structure, was more ordered in the complex, forming two anti-parallel β-strands (Fig. 5*A*). In the c-di-AMP·PstA_SA_ complex structure, a salt bridge is formed between the highly conserved amino acids Asp-12 in α-helix 1 and Arg-67 at the beginning of the B′-loop (Fig. 5, *A* and *C*). In the apo structure no salt bridge is observed, as only density for the C_β_ is present for Arg-67 (Fig. 5*C*).

**FIGURE 6.**
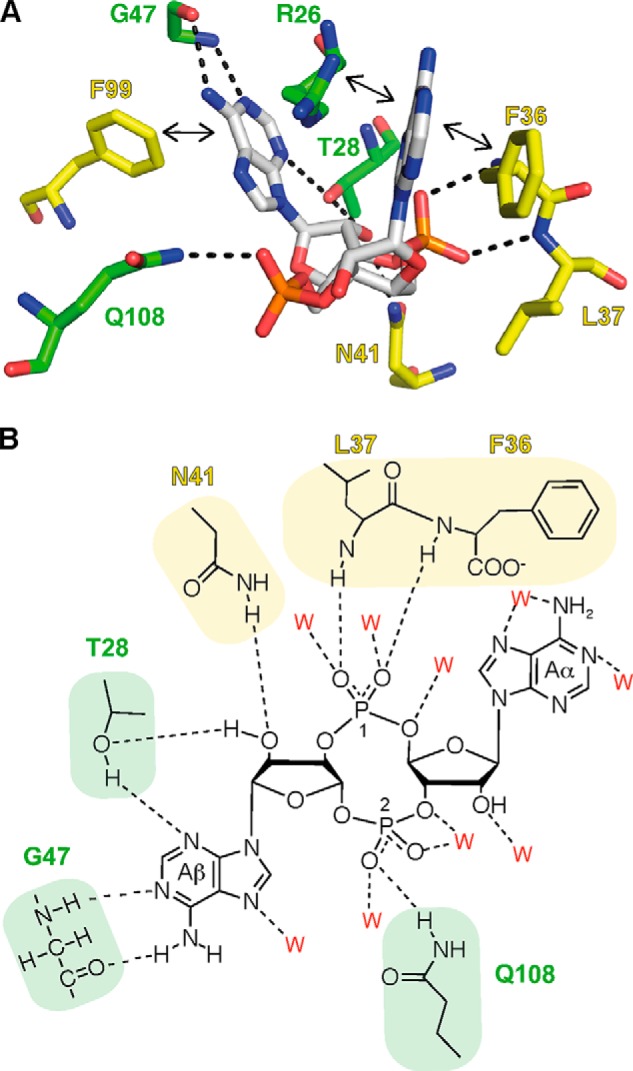
**c-di-AMP/PstA_SA_ hydrogen bonding network.** *A,* c-di-AMP-binding site with amino acid residues from monomer A shown in *green* and monomer B shown in *yellow*. Residue Phe-36 forms a face-to-face π-stacking with adenine, and the other residues Leu-37, Asn-41 (monomer B), Thr-28, Gly-47, and Gln-108 (monomer A) form hydrogen bonds with different parts of the c-di-AMP ligand. Additional residues shown in the structure are Phe-99 (monomer B) and Arg-26 (monomer A), forming an edge-to-face π-stacking and a cation-π interaction, respectively. Hydrogen bond interactions are indicated with *dotted lines* and π interactions with *double-head arrows. B,* schematic representation of the hydrogen bond interactions between c-di-AMP-binding site residues. Residues from monomer A are shown in *green* and from monomer B in *yellow*. The water molecules (indicated as *red W*) forming hydrogen bonds are also shown in this schematic diagram. The details of these interactions are described under “Results,” and adenines are labeled Aα and Aβ, and PO_4_ groups 1 and 2, and will be referred to as such in the text.

Clear density for the c-di-AMP ligand was visible at each monomer-monomer interface within the PstA_SA_ trimer (Fig. 5*D*). The c-di-AMP-binding mode at the three monomer-monomer interfaces is equivalent, and therefore only the interaction at the monomer A-monomer B interface will be discussed. The c-di-AMP-binding site is overall positively charged due to residues Asn-24, Asn-41, and Gln-108, with the latter two residues being in hydrogen bonding distance with the ligand (Fig. 5*E*). Both phosphate groups of the ligand form oxy-anion holes; one such anion-stabilizing hydrogen-bonding pocket is formed by the interaction of the PO_4_ group 1 with two water molecules and the backbone amides of Phe-36 and Leu-37 (monomer B), whereas another is formed by the PO_4_ group 2 through hydrogen bonding with Gln-108 and two water molecules (Fig. 6). The adenine ring Aα is held in place by a face-to-face π stacking interaction with Phe-36 (monomer B) and a π-cation interaction with Arg-26 (monomer A), whereas the adenine ring Aβ forms a face-to-edge stacking interaction with Phe-99, and three hydrogen bonds with the amide and carbonyl group of Gly-47 (monomer A) and the side chain of Thr-28 (monomer A). Asn-41 forms a hydrogen bond with the ribose ring, and additional hydrogen bonds are formed between surrounding waters, which are conserved across three monomers, and the oxygen and nitrogen groups of the ribose and adenine rings, respectively (Fig. 6). It is notable that although each monomer provides approximately half a ligand-binding site, the interactions with the ligand are very different in each half-binding site.

##### Mutagenesis Analysis Confirms Essential Function of Conserved c-di-AMP-interacting Residues

**FIGURE 7.**
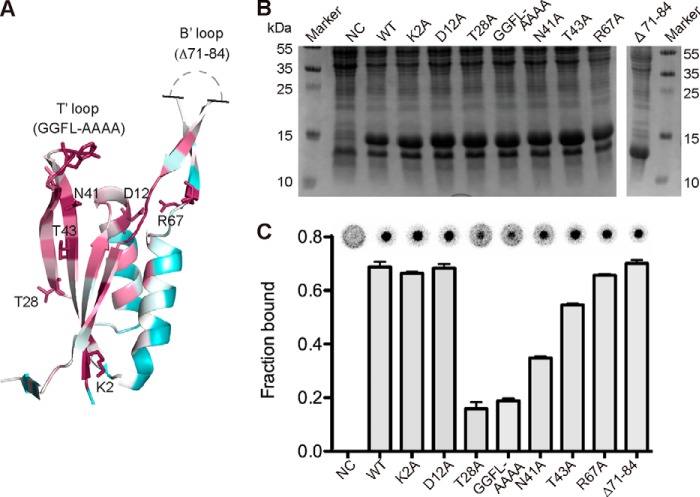
**Location of highly conserved amino acids in PstA_SA_ and their role in c-di-AMP binding.** *A,* schematic representation of the PstA_SA_ monomer from the complex structure in the ConSurf representation (*purple*, high conservation; *green,* medium conservation; *white,* low conservation) with the mutated amino acids shown in *stick* representation. Unstructured region of the B′-loops is schematically indicated. *B,* Coomassie-stained gel of whole cell extracts prepared from *E. coli* BL21(DE3) strains containing the empty vector pET28b as negative control (*NC*), strains overproducing His-PstA_SA_ (*WT*), or different protein variants containing the amino acid substitutions as indicated *above* each lane. A protein marker was run alongside, and the molecular mass of the marker proteins is indicated in kDa on the *left* and *right sides* of the panel. *C,* c-di-AMP binding assays. DRaCALAs were performed with ^32^P-labeled c-di-AMP and the *E. coli* whole cell lysates described in *B*. Representative DRaCALA spots are shown, and the fraction bound was calculated as described previously, and the average values and standard deviation of two independent experiments are plotted.

**FIGURE 8.**
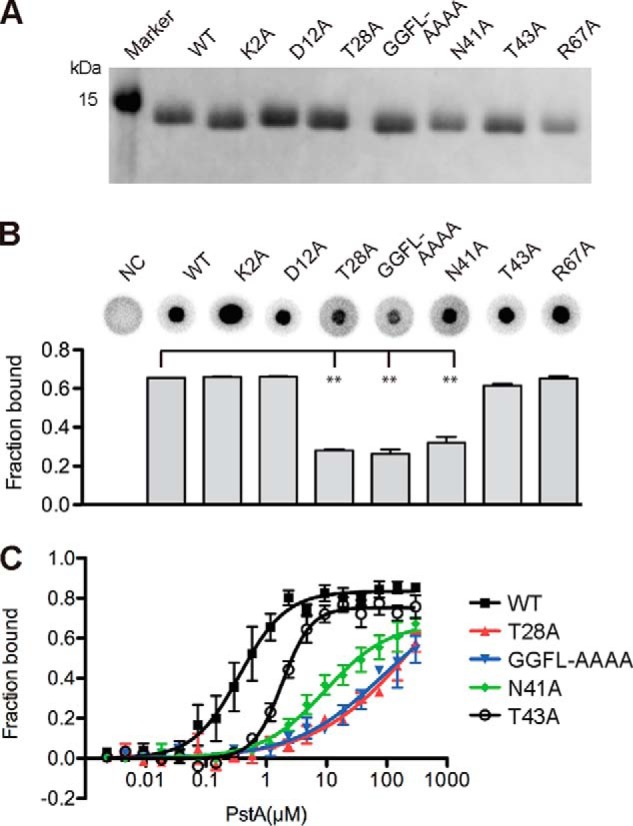
**Identification of PstA variants with c-di-AMP-binding defects.** *A,* Coomassie-stained gel with purified His-PstA_SA_ protein (*WT*) and different protein variants containing the amino acid substitutions as indicated *above* each lane. *B,* c-di-AMP binding assays. DRaCALAs were performed with ^32^P-labeled c-di-AMP and purified His-PstA_SA_ protein (*WT*) or variants with the indicated amino acid substitution. The proteins were used at a concentration of 10 μm, and as negative control (*NC*) no protein was added to the binding assays. Representative DRaCALA spots are shown, and the fraction bound was calculated as described previously, and the average values and standard deviation of two independent experiments were plotted. *C,* binding curve and *K_d_* determination for c-di-AMP and purified His-PstA_SA_ or protein variants with the indicated amino acid substitutions. *K_d_* values were determined from the curve as described previously (41).

The sequence analysis (Fig. 1*A*) and the c-di-AM·PstA_SA_ complex structure (Figs. 5 and 6) highlighted several conserved residues involved in c-di-AMP binding. To confirm the physiological relevance of the c-di-AMP-binding site observed in the complex structure, a mutagenesis analysis was performed. Specifically, the conserved amino acids Thr-28 and Asn-41 mutated to alanines as well as the GGFL amino acids (residues 34–37) that form the small T′-loop of PstA proteins were simultaneously mutated to alanines. Variants with these mutations were expected to be compromised in c-di-AMP binding as these residues make direct contact with the ligand (Fig. 6). As control, other highly conserved residues Lys-2 and Thr-43 and residues Asp-12 and Arg-67 (forming a salt bridge upon ligand binding but not in direct contact with the ligand) were mutated to alanines (Figs. 5*A* and 7*A*). Finally, a variant with a deletion of the large B′-loop, which distinguishes PstA proteins from classical PII proteins but is not expected to be involved in c-di-AMP binding, was constructed (variant Δ71–87 with deletion of amino acids 71–87) (Fig. 7*A*). The His-PstA_SA_ protein and the different His-PstA_SA_ variants K2A, D12A, T28A, N41A, T43A, GGFL-AAAA, R67A, and Δ71–87 were overexpressed in *E. coli*; whole cell lysates were prepared, and c-di-AMP binding was assessed by DRaCALAs as described previously (Fig. 7, *B* and *C*) (9). Reduced binding was seen for His-PstA_SA_ variants T28A, N41A, and GGFL-AAAA, although the binding was not markedly affected for variants K2A, D12A, T43A, R67A, and Δ71–87 (Fig. 7, *B* and *C*). To confirm this, the different variants were purified and used in DRaCALAs (Fig. 8). The His_6_-PstA_SA_Δ71–87 protein precipitated during our standard purification process and could not be further analyzed. However, the protein showed normal binding to c-di-AMP using whole cell lysates, indicating that the large B′-loop was not involved in c-di-AMP binding. Identical c-di-AMP binding results were obtained for the purified His-PstA_SA_ variants as with whole cell lysates, with variants T28A, N41A, and GGFL-AAAA showing a binding defect (Fig. 8*B*). Next, binding affinities were determined with the purified proteins. Initial binding studies showed that c-di-AMP bound to variants His-PstA_SA_ K2A, D12A, and R67A with *K_d_* values of 0.34 ± 0.04, 0.32 ± 0.05, and 0.48 ± 0.04 μm, which are similar to that of wild type His_6_-PstA_SA_. A 5-fold increase in the *K_d_* value was observed for the T43A variant, to which c-di-AMP bound with a *K_d_* value of 1.8 ± 0.2 μm as compared with wild type His-PstA_SA_ with a *K_d_* value of 0.37 ± 0.03 μm; however, the binding still reached saturation at a protein concentration above 20 μm (Fig. 8*C*). The protein variants with the least binding capacity were the N41A variant with a *K_d_* of 9.6 ± 2.3 μm and the T28A and GGFL-AAAA variants, for which no *K_d_* values could be determined as the binding did not reach saturation even at a protein concentration of 300 μm. Therefore, in agreement with interactions seen in the co-complex structure, conserved residues, Thr-28, Asn-41, and the T′-loop residues GGFL are important for c-di-AMP binding. Although other highly conserved residues, including residues Asp-12 and Arg-67, which form a salt bridge in the ligand-bound structure, are dispensable for c-di-AMP binding. These data suggest that the physiologically relevant c-di-AMP-binding site was revealed in the complex structure.

## DISCUSSION

**FIGURE 9.**
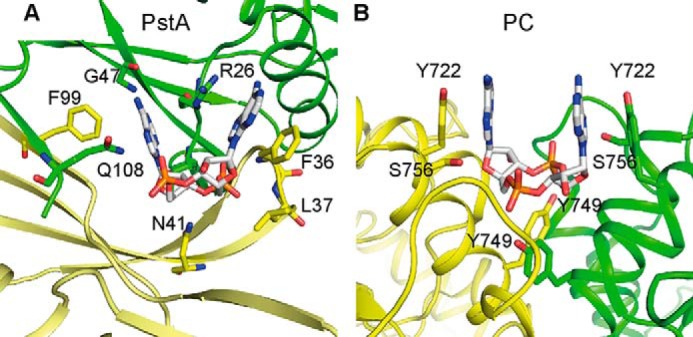
**Comparison of *S. aureus* PstA and *L. monocytogenes* PC c-di-AMP-binding sites.** *A* and *B*, schematic representations of c-di-AMP-binding site in *S. aureus* PstA_SA_ (*A*) and *L. monocytogenes* PC (PDB 4QSH) (11) (*B*) with different monomers shown in *green* and *yellow*, respectively. Amino acid residues forming key contact with the ligand are labeled and shown as *sticks*.

Several c-di-AMP receptor proteins have now been identified (9–11, 43); however, so far only the structure of the *L. monocytogenes* pyruvate carboxylase (PC) has been solved in complex with c-di-AMP (11). Here, we present the structure of the second target protein, namely the *S. aureus* PII-like signal transduction protein PstA, in complex with c-di-AMP (Figs. 5 and 6). As discussed below, although the residues involved in nucleotide binding are very distinct between these two proteins (Fig. 9) and a classical consensus sequence cannot be established, common features for c-di-AMP-binding sites can now be proposed. In addition, a comparative analysis of the apo and complex structures allows us to present a model on how this protein functions as a signal transduction protein.

Although PstA is annotated as PII-like signal transduction protein, our structural analysis together with a detailed bioinformatics analysis suggest that PstA_SA_ is a prototype of a new protein family with clearly distinct features from classical PII proteins. This new family of proteins currently contains around 900 members in the nonredundant protein database (Figs. 1*A* and 3*A*). PstA homologs are present mainly in the Firmicutes, with some homologs present in green non-sulfur bacteria, Mycoplasmas, Spirochetes, and other unknown/unclassified bacteria. This is consistent with the identification of predicted c-di-AMP cyclases among most bacteria belonging to the Firmicutes group. However, not all bacteria that can produce c-di-AMP possess PstA homologs. Notably, a PstA homolog is present in *Bacillus subtilis* 168 but absent in the pathogenic strains *Bacillus cereus*, *Bacillus anthracis,* and *B. thuringiensis*. Conversely, the thermophilic bacteria *Sphaerobacter thermophilus* DSM 20745 and *Thermomicrobium roseum* DSM 5159 encode three PstA orthologs, whereas the majority of other bacteria contained only one PstA-type protein.

Comparison of the crystal structures of PII proteins and PstA revealed that they share the same core fold. However, as shown here, the two main loops, named T- and B-loops in PII proteins and T′- and B′-loops in PstA, are reversed in length and as we would speculate also in function. In PII proteins, the B-loop is short and contains the conserved Walker A motif sequence TG*XX*GDGKI required for ADP or ATP binding, whereas in PstA the short T′-loop contains the GGFL residues, which are part of the highly conserved TKL*XXX*GGFL*XX*GNTT motif, which is critical for c-di-AMP binding. The large T-loop in PII proteins is involved in protein-protein interaction and changes its conformation upon ligand binding. Two highly conserved PII protein residues at the base of the large T-loop, Gln-39 and Lys-58, interact with 2-OG when it is at a high cellular concentration, and therefore bound to the protein, or form a salt bridge when the cellular concentration is low and the protein is in an unbound state. This results in a conformational change in the T-loop allowing it to interact or disengage from its effector protein, thus providing a structure-based mechanism for the signal transduction properties of PII proteins in response to 2-OG levels. By obtaining the PstA apo- and c-di-AMP complex structures, we can now propose a structure-based model of how PstA functions as a signal transduction protein. In the apo-PstA structure both the T′- and B′-loops were unstructured, indicating that they are highly flexible in the nucleotide-free state (Fig. 3). Upon c-di-AMP binding, the T′-loop becomes stabilized and structured through interactions with the nucleotide. The nucleotide-induced ordering of the T′-loop results in small structural perturbations throughout the PstA trimer, and in the complex structure a salt bridge between Asp-12 at the end of α-helix 1 and Arg-67 at the beginning of the B′-loop was observed (Fig. 5). In the apo structure, the side chain of Arg-67 is not completely visible in the electron density maps suggesting that it is highly flexible (Fig. 5*C*). We speculate that the formation of the Asp-12–Arg-67 salt bridge could be associated with the stabilization of the B′-loop, which forms two anti-parallel β-strands that protrude from each monomer in the trimer (Fig. 5*B*). However, further work is needed in the future to experimentally test this hypothesis. As shown in this study, neither residue Asp-12 nor Arg-67 is essential for c-di-AMP binding (Figs. 7 and 8); however, both residues are highly conserved within the PstA proteins indicating a functional importance (Fig. 1). By analogy to PII proteins where residues Gln-39 and Lys-58 stabilizes the conformation of the T-loop, it can be speculated that residues Asp-12 and Arg-67 might play a similar role in stabilizing the B′-loop in PstA proteins. Again, similar to PII proteins, the conformational change in the large B′-loop would presumably provide an interaction surface for downstream effector proteins and in this manner affect their activity. In the absence of c-di-AMP, such interactions would be disengaged or prevented, thus establishing an elegant nucleotide-dependent signaling cascade. However, currently neither the cellular function of the *S. aureus* PstA protein nor any other PstA homologs are known nor have any interaction partner of PstA proteins been identified. It is notable that the B′-loop displays large variations among PstA homologs (Fig. 1*A*), and this might allow different members of this protein family to interact with diverse classes of effector proteins, similar to classical PII proteins.

The apo-PstA structure was solved using the deposited *P. pentosaceus* ATCC 25745 protein PEPE_1480 structure as a model, which belongs to the same protein family. In the *Pediococcus* structure, the T′-loop and a large part of B′-loop are structured, which we would now refer to as being in the “ligand-bound state.” This state was observed even though the protein crystals were grown in the absence of c-di-AMP. However, the crystallization buffer contained 0.2 m Li_2_SO_4_ and a closer inspection of the *Pediococcus* structure revealed the presence of a sulfate molecule in a similar position to one of the phosphate groups of the c-di-AMP ligand in the PstA·nucleotide complex. Hydrogen bonds are formed between the sulfate group and a conserved arginine residue (corresponding to Arg-26 in PstA_SA_) and the amide group of the highly conserved T′-loop phenylalanine residue (Phe-36 in PstA). These interactions could mimic the ligand-bound state by stabilizing the T′-loop.

The only other structure of a c-di-AMP bound protein complex is that of the *L. monocytogenes* PC enzyme. This enzyme is a tetramer composed of two layers of dimers with two nucleotide-binding sites (11). The c-di-AMP-binding sites are at symmetrical monomer-monomer interfaces, with the same set of amino acids from each monomer providing a half-binding site, consistent with the 2-fold symmetric c-di-AMP nucleotide (Fig. 9) (11). Specifically, a tyrosine residue from each monomer provides π-stacking interaction with the adenine rings, and other tyrosine and serine residues form hydrogen bonds with the terminal oxygen atoms of the phosphate groups (Fig. 9) (11). The binding mode observed in the *Listeria* enzyme is similar to the previously reported binding of c-di-GMP with the eukaryotic immune receptor protein STING (44–46). However, no clear specificity determinants can be observed for preferential interaction of the PC enzyme of *L. monocytogenes* with c-di-AMP *versus* other cyclic dinucleotides such as c-di-GMP. As the PstA protein is trimeric (Figs. 2 and 5), this results in an inherent asymmetry of the binding site with different amino acids from each monomer contributing to ligand binding (Figs. 6 and 9). The bases of nucleotide ligands often form face-to-face π-stacking interactions with aromatic amino acids in the receptor. In the c-di-AMP·PstA complex, however, only one of the adenine bases (Aα) forms a face-to-face π-stacking with Phe-36, located within the highly conserved T′-loop, whereas the second adenine (Aβ) forms a face-to-edge stacking interaction with Phe-99; both of these amino acids are on the same monomer. In addition to the face-to-edge stacking interaction, the second adenine base (Aβ) forms additional hydrogen bond interactions, including between its amino group and the amide and carbonyl groups of Gly-47 (Figs. 6 and 9). The amino group in the adenine base would be replaced with a carbonyl group in a guanine base, and therefore such an interaction would be energetically unfavorable. In addition, the amino group in a guanine base would clash with the amide backbone of Leu-35, thus providing a rationale for the specific binding of c-di-AMP to PstA.

By comparing the PstA and PC ligand-bound structures, it is apparent that c-di-AMP-binding sites are not conserved at the amino acid sequence level; thus, establishing a universal sequence motif for predicting c-di-AMP-binding sites in other proteins may not be possible (Fig. 9). Despite this, a number of conserved features of c-di-AMP-binding sites can now be identified, including a surface-accessible binding pocket formed between two protomers, with an overall positive charge (as observed for both PC and PstA), aromatic amino acids, such as tyrosine or phenylalanine (at least one in asymmetric binding sites and likely two in symmetric binding site), and additional conserved amino acids, such as threonine, serine, asparagine, or glutamine, which can provide for hydrogen bond interactions with the phosphate and ribose groups. Therefore, our study not only provides insight into how members of the PstA protein family might function as signal transduction proteins, but also highlights conserved features of c-di-AMP-binding sites that will aid future studies and allow for a more rational approach for identifying c-di-AMP-binding sites within target proteins.

## Footnotes

4 The abbreviations used are:

c-di-AMP: cyclic diadenylate monophosphate
SEC-MALS: size exclusion multiangle laser light scattering
2-OG: 2-oxoglutarate
DRaCALA: differential radial capillary action of ligand assay
PDB: Protein Data Bank
PC: pyruvate carboxylase.
